# Disruption of PTH Receptor 1 in T Cells Protects against PTH-Induced Bone Loss

**DOI:** 10.1371/journal.pone.0012290

**Published:** 2010-08-20

**Authors:** Hesham Tawfeek, Brahmchetna Bedi, Jau-Yi Li, Jonathan Adams, Tatsuya Kobayashi, M. Neale Weitzmann, Henry M. Kronenberg, Roberto Pacifici

**Affiliations:** 1 Division of Endocrinology, Metabolism and Lipids, Department of Medicine, Emory University, Atlanta, Georgia, United States of America; 2 Immunology and Molecular Pathogenesis Program, Emory University, Atlanta, Georgia, United States of America; 3 Endocrine Unit, Massachusetts General Hospital, Boston, Massachusetts, United States of America; 4 Atlanta VA Medical Center, Decatur, Georgia, United States of America; New York University, United States of America

## Abstract

**Background:**

Hyperparathyroidism in humans and continuous parathyroid hormone (cPTH) treatment in mice cause bone loss by regulating the production of RANKL and OPG by stromal cells (SCs) and osteoblasts (OBs). Recently, it has been reported that T cells are required for cPTH to induce bone loss as the binding of the T cell costimulatory molecule CD40L to SC receptor CD40 augments SC sensitivity to cPTH. However it is unknown whether direct PTH stimulation of T cells is required for cPTH to induce bone loss, and whether T cells contribute to the bone catabolic activity of PTH with mechanisms other than induction of CD40 signaling in SCs.

**Methodology/Principal Findings:**

Here we show that silencing of PTH receptor 1 (PPR) in T cells blocks the bone loss and the osteoclastic expansion induced by cPTH, thus demonstrating that PPR signaling in T cells is central for PTH-induced reduction of bone mass. Mechanistic studies revealed that PTH activation of the T cell PPR stimulates T cell production of the osteoclastogenic cytokine tumor necrosis factor α (TNF). Attesting to the relevance of this effect, disruption of T cell TNF production prevents PTH-induced bone loss. We also show that a novel mechanism by which TNF mediates PTH induced osteoclast formation is upregulation of CD40 expression in SCs, which increases their RANKL/OPG production ratio.

**Conclusions/Significance:**

These findings demonstrate that PPR signaling in T cells plays an essential role in PTH induced bone loss by promoting T cell production of TNF. A previously unknown effect of TNF is to increase SC expression of CD40, which in turn increases SC osteoclastogenic activity by upregulating their RANKL/OPG production ratio. PPR-dependent stimulation of TNF production by T cells and the resulting TNF regulation of CD40 signaling in SCs are potential new therapeutic targets for the bone loss of hyperparathyroidism.

## Introduction

Parathyroid hormone (PTH) is a major regulator of calcium metabolism which defends against hypocalcemia, in part by stimulating bone resorption and thereby the release of calcium from the skeleton. Chronic overproduction of PTH is a cause of skeletal and extra-skeletal disease. Secondary hyperparathyroidism has been implicated in the pathogenesis of senile osteoporosis [1], while primary hyperparathyroidism (PHP), is associated with accelerated bone loss [2], osteopenia [3], [4], [5], and increased bone turnover [3], an independent risk factor for fractures. Furthermore, PHP is a cause of extra-skeletal manifestations stemming from increased bone resorption such as hypercalcemia, recurrent nephrolithiasis, renal failure, and mental changes [4].

PHP and secondary hyperparathyroidism are mimicked by cPTH infusion. By contrast, daily injections of PTH, a regimen known as intermittent PTH (iPTH) treatment, cause a marked stimulation of bone formation and increase bone strength [6]. As a result, iPTH is an approved treatment modality for osteoporosis [7].

Hyperparathyroidism and cPTH treatment cause cortical bone loss by enhancing endosteal resorption through stimulation of osteoclast (OC) formation and activity [4], [8], [9], and often lead to trabecular bone loss [4], [8], although mild PHP and cPTH treatment of young mice may induce a modest increase in cancellous bone [3], [5], [10], [11]. Hyperparathyroidism and cPTH treatment increase bone turnover in trabecular and cortical bone, as evidenced by elevations in histomorphometric and biochemical markers of resorption and formation [8], [12], [13].

The effects of PTH on bone result from its binding to the PTH/PTH-related protein (PTHrP) receptor (PPR or PTHR1), expressed on bone marrow (BM) stromal cells (SCs), OBs and osteocytes [13], [14], [15]. The catabolic effect of PTH has been shown to be mediated, in part, by enhanced production of receptor activator of nuclear factor-κB ligand (RANKL) and macrophage colony stimulating factor (M-CSF), and decreased production of osteoprotegerin (OPG) by SCs and OBs [16], [17]. Furthermore, additional BM cells contribute to the catabolic activity of PTH in vivo. Among them are T cells, a lineage that expresses PPR [18], [19], [20] and responds to PTH [21], [22]. T cells also express surface receptors that bind to, and activate counter-receptors found on the surface of cells of the osteoblastic lineage [23]. One of these osteoblastic cell surface receptors is CD40, which upon ligation by T cell expressed CD40 ligand (CD40L), provides survival and proliferative cues to SCs and OBs [23].

A role for T cells in the response to PTH was first suggested by Hory et al [24], who reported that transplantation of human parathyroid gland fragments from patients with primary and secondary hyperparathyroidism into nude mice fails to stimulate OC formation and bone resorption. We have further reported that cPTH fails to induce OC formation, bone resorption and cortical bone loss in mice lacking T cells [11]. We also found that PTH-induced bone loss is blocked in WT mice by treatment with T cell-depleting antibodies (mAbs) [11]. Our studies further disclosed the existence of a cross-talk between T cells and SCs mediated by the CD40L/CD40 signaling system. T cells expand the pool of BM SCs and sensitize SCs to PTH through CD40L. As a result, the absence of T cells or deletion of T cell expressed CD40L blunts the bone catabolic activity of PTH by decreasing BM SC number, their RANKL/OPG ratio, and hence their osteoclastogenic activity [11].

Further studies from our laboratory have also linked T cells to the bone anabolic activity of iPTH, although the involved mechanisms are unrelated to CD40L. Treatment with iPTH increases the CD8+ T cell production of Wnt10b, a Wnt ligand that stimulates osteoblastogenesis by activating Wnt signaling in SCs and OBs. As a result, the bone anabolic activity of iPTH is markedly reduced in T cell-deficient mice and in mice with a selective disruption of T cell Wnt10b production [19].

In spite of the growing evidence supporting a role for T cells in the mechanism of cPTH actions on bone, the mechanisms involved are only partly understood, as it remains unknown whether cPTH acts directly on T cells to exert its effects in bone. Also unknown is whether T cells contribute to the bone catabolic activity of cPTH via mechanisms other than induction ofCD40 signaling in SCs. To address these issues we have conditionally silenced PPR in T cells and determined whether PTH activation of PPR in T cells is required for cPTH to exert its bone catabolic activity. We show that blocking PPR signaling in T cells abrogates the capacity of cPTH treatment to stimulate OC formation and induce bone loss. We further show that PPR signaling in T cells stimulates the secretion of the osteoclastogenic cytokine TNF, a factor which promotes OC differentiation and bone resorption both directly, and by regulating the production of RANKL and OPG by SCs and OBs. Therefore, direct targeting of T cells by PTH is required for PTH to induce its bone catabolic effects, and T cell production of TNF is a novel mechanism by which T cells contributes to the bone catabolic activity of cPTH.

## Results

### Generation of PPR^T cells^ −/− mice

To elucidate whether direct stimulation of T cells by cPTH through PPR signaling is required for cPTH to induce bone loss, we generated a mouse, referred to herein as PPR^fl/fl^/Lck-Cre, or PPR^T cells^ −/− mouse, with targeted deletion of PPR in αβ and γδ T cells. To this end, C57BL/6 mice harboring a PPR allele with floxed exon E1 (PPR^fl/fl^) developed by Kobayashi et al [25] were crossed with C57BL/6 Lck-Cre transgenic mice, a strain that expresses the Cre recombinase in the early stages (DN2-DN3) of thymocyte development [26]. Thus, in the PPR^T cells^ −/− mouse the PPR gene is silenced in T cell precursors and mature αβ and γδ T cells.

**Figure 1:**
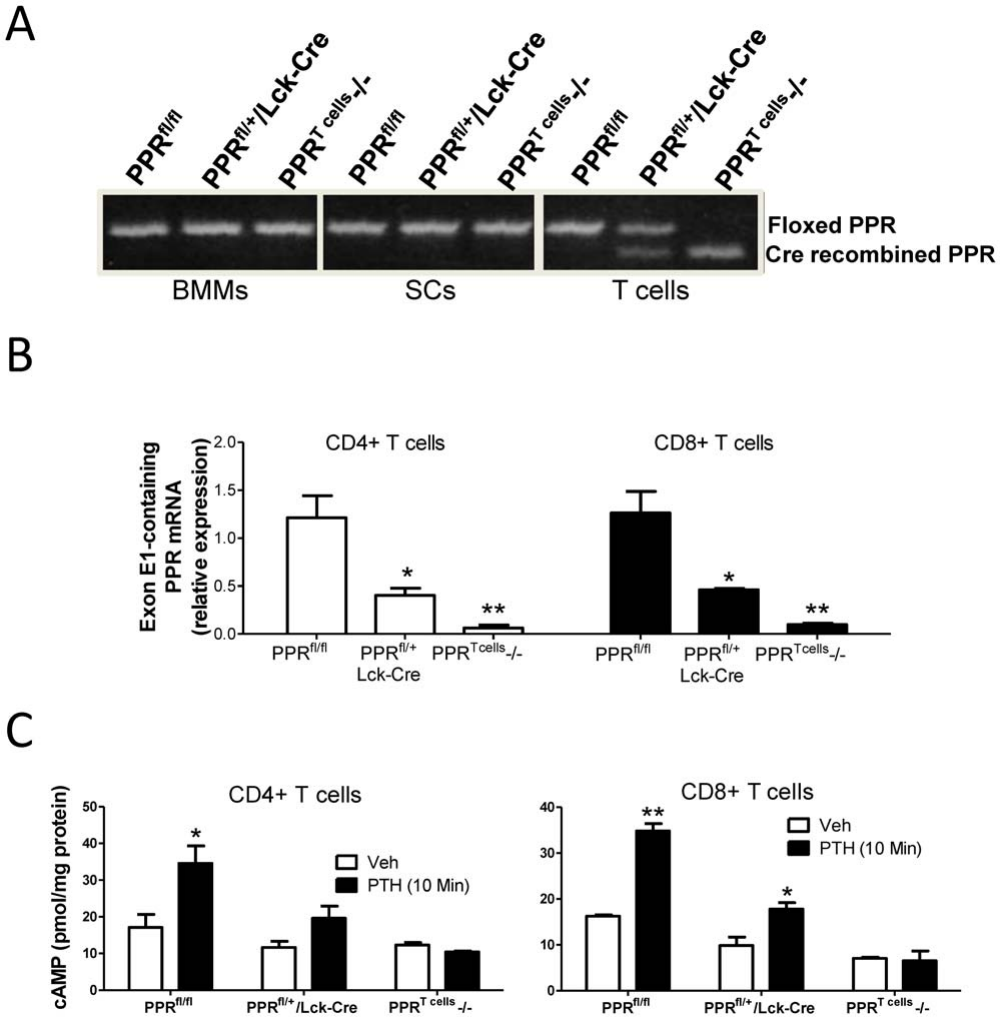
Confirmation of PPR gene disruption in PPR^T cells^ −/− mice. **A** Characterization of floxed and Cre recombined PPR alleles in BMMs, SCs and T cells from PPR^T cells^ −/− and control mice. **B** PPR mRNA levels (Mean ± SEM) in CD4+ and CD8+ T cells from PPR^T cells^ −/− and control mice. * = p<0.05 and ** = p<0.01 compared to the corresponding PPR^fl/fl^ group. **C** Effect (Mean ± SEM) of in vitro PTH treatment (50 nM) on cAMP accumulation by splenic CD4+ and CD8+ T cells from PPR^T cells^ −/− and control mice. * = p<0.05 and ** = p<0.01 compared to the corresponding vehicle group.

PCR Analysis of DNA from BM macrophages (BMMs) and SCs revealed the presence of a full length PPR gene in all genotypes, ensuring absence of Cre expression in these cells (Fig. 1a). Conversely, analysis of T cell DNA revealed that partial or complete recombination of the PPR gene occurred in PPR^fl/+^/Lck-Cre and PPR^fl/fl^/Lck-Cre mice, respectively, due to the successful deletion of floxed exon E1. Quantitative analysis of PPR RNA by real time RT-PCR, confirmed deletion (>90%) of the floxed exon E1 of the PPR gene in spleen CD4+ and CD8+ T cells from PPR^T cells^ −/− mice, as compared to CD4+ and CD8+ cells from PPR^fl/fl^ mice (Fig. 1b). Moreover, in vitro PTH treatment induced a significant increase in the production of cAMP in spleen CD4+ and CD8+ T cells from PPR^fl/fl^ control mice, but not in those from PPR^T cells^−/− mice (Fig. 1c). These findings demonstrate that T cell expression of functioning PPR is specifically blocked in PPR T cells −/− mice as a result of efficient Cre-mediated exon E1 ablation.

At 6 weeks of age PPR^T cells^ −/− mice and control littermates had no gross skeletal alterations, and similar femoral (bone mineral density) BMD values. All strains had similar serum levels of calcium, phosphate and PTH (Figure S1). Moreover, all mice had comparable numbers of splenic CD4+ and CD8+ T cells, a similar expression of the activation marker CD25, and comparable T cell proliferation.

### cPTH treatment fails to cause bone loss, stimulate bone resorption and induce in vitro OC formation in mice lacking PPR signaling in T cells

Infusion of hPTH 1–34 at the rate of 80 µg/kg/day for 2 weeks, a treatment modality referred to hereafter as cPTH, models the steady state elevation of PTH levels characteristic of moderate to severe PHP [11]. To determine whether PPR signaling in T cells is required for cPTH to induce bone loss, male PPR^T cells^ −/− and littermate controls were infused with vehicle or cPTH starting at 16 weeks of age, a time point when C57/BL6 mice reach peak bone size and mechanical strength [27], and cPTH infusion results in the loss of cortical and trabecular bone. By contrast, treatment with cPTH at the age of 10 weeks results in cortical but not trabecular bone loss [11].

**Figure 2:**
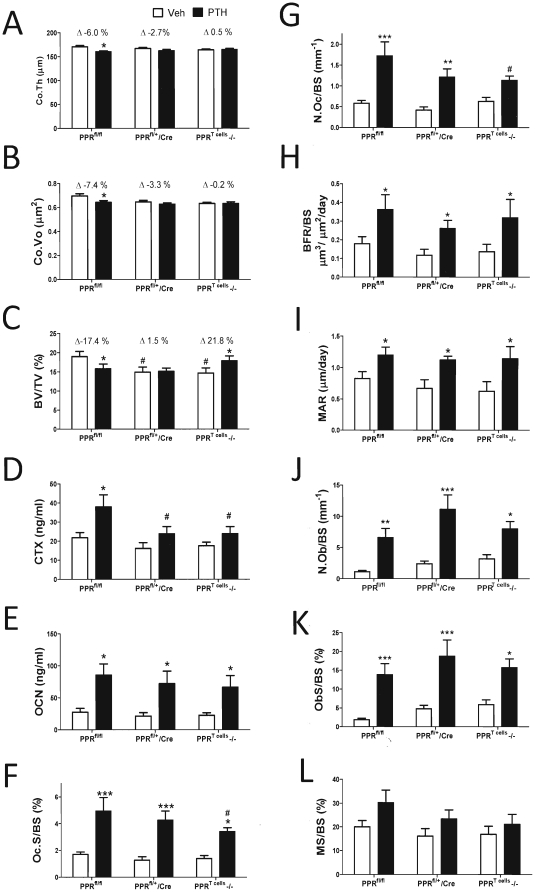
Effects of cPTH treatment on indices of bone structure and turnover in PPR^T cells^ −/− and control mice. **A**. µCT analysis of femur cortical bone thickness (Co.Th). **B**. µCT analysis of femur cortical bone volume (Co.Vo). **C**. µCT analysis of trabecular bone volume (BV/TV). **D** Analysis of serum CTX levels by ELISA. **E** Analysis of serum OCN levels by ELISA. **F–L** Histomorphometric analysis femoral trabecular bone. F: percentage of bone surface covered by osteoclasts (Oc.S/BS). G: number of osteoclasts per mm bone surface (N.Oc/BS). H: Bone formation rate (BFR). I: Mineral Apposition rate (MAR), J: Percentage of bone surface covered by osteoblasts (Ob.S/BS). K: Number of osteoblasts per mm bone surface (N.Ob/BS). L: percentage of mineralized bone surface (MS/BS). * = p<0.05, ** = p<0.01 and *** = p<0.001 compared to the corresponding vehicle treated group. # = p<0.05 compared to the corresponding PPR^fl/fl^ mice. n = 18–22 mice per group for µCT and serum measurements. n = 10–12 mice per group for bone histomorphometry. Data are means ± SEM.

To assess the differential effects of cPTH on cortical and trabecular bone, micro-computed tomography (µCT) was utilized to analyze femurs harvested at sacrifice. All groups of vehicle treated mice had similar values of cortical thickness and cortical volume. Treatment with cPTH induced significant cortical thinning and loss of cortical volume in PPR^fl/fl^ mice (Fig. 2a,b). In contrast, cPTH did not cause significant cortical bone loss in heterozygous PPR^fl/+^/Lck-Cre and homozygous PPR^T cells^ −/− mice. Vehicle treated PPR^fl/+^/Lck-Cre and PPR^T cells^ −/− mice had lower BV/TV as compared to vehicle treated PPR^fl/fl^ mice, suggesting that PPR signaling in T cells may contribute to the anabolic activity of endogenous PTH. Treatment with cPTH led to a significant decrease in trabecular BV/TV in PPR^fl/fl^ mice but not in PPR^fl/+^/Lck-Cre mice. Strickingly, treatment of PPR^T cells^ −/− mice with cPTH resulted in a significant increase in BV/TV (Fig. 2c).

Measurements of serum C-terminal telopeptide of collagen (CTX), a biochemical marker of resorption, revealed that cPTH induced a significant increase in bone resorption in PPR^fl/fl^ mice, but not in PPR^fl/+^/Lck-Cre and PPR^T cells^ −/− mice (Fig. 2d). Assessment of osteocalcin (OCN) levels, a marker of bone formation, showed that serum osteocalcin was equally increased by cPTH in all groups (Fig. 2e).

Histomorphometric analysis of femoral trabecular bone revealed that the number of OCs per bone surface (NOc/BS) and the OC surface per bone surface (Oc.S/BS), two indices of bone resorption, were increased by cPTH to a greater amount in control mice than in PPR^T cells^ −/− mice (Fig. 2f,g). In contrast, bone formation rate (BFR/BS), mineral apposition rate (MAR), the numbers of OBs per bone surface (N.Ob/BS), and the percent of bone surface covered by OBs (Ob.S/BS), which are indices of bone formation, were significantly augmented by cPTH in all groups (Fig. 2h–k). The changes in the percentage of mineralized surface, another index of bone formation, did not reach statistical significance in any of the groups (Fig. 2l). Thus, while PPR signaling in T cells is required for cPTH to induce bone loss and stimulate bone resorption, it is not required for cPTH to stimulate bone formation. To exclude the possibility of a sexually dimorphic role of PPR signaling in T cells, a second experiment was conducted using female mice of 16 weeks of age. Treatment with cPTH induced significant cortical and trabecular bone loss in PPR^fl/fl^, while it had no effect in PPR^T cells^ −/− mice (Figure S2). Mirroring the effects induced on bone volume, cPTH caused an increase in histomorphometric and biochemical indices of bone resorption in PPR^fl/fl^ and PPR^fl/+^/Lck-Cre mice, but not in PPR^T cells^ −/− mice. In contrast, cPTH increased histomorphometric and biochemical indices of bone formation in all strains. Thus, PPR signaling in T cells is required for cPTH to cause bone loss in male and female mice.

Ovariectomy (ovx) and cPTH are known to have additive bone catabolic effects, and previous studied from our laboratory have implicated T cells in the mechanism of ovx induced bone loss [28]. We thus investigated whether silencing of PPR in T cells prevents bone loss in mice subjected to ovx and cPTH treatment. In vivo measurements of femoral BMD by (Dual X-ray Absorptiometry) DEXA revealed that ovx causes equal bone loss in vehicle treated PPR^Tcells^ −/− and control mice. Measurements DEXA and µCT further revealed that ovx PPR^fl/fl^ mice treated with cPTH sustained a greater cortical and trabecular bone loss than ovx PPR^fl/fl^ mice treated with vehicle. By contrast cPTH did not induce significant further bone loss in ovx PPR^T cells^ −/− mice (Figure S3), thus confirming that PPR signaling in T cells plays a critical role in the mechanism of action of cPTH.

### PPR signaling in T cells increases the expression of CD40 and osteoclastogenic cytokines in SCs

**Figure 3:**
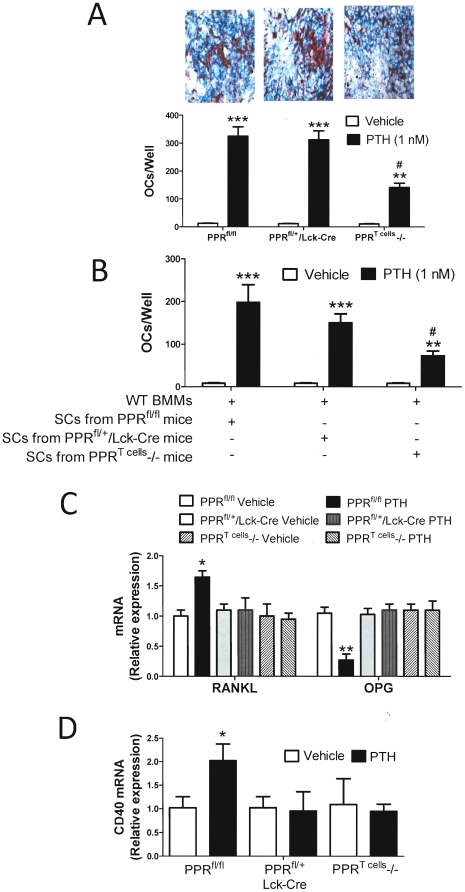
PTH effects on OC formation, SC osteoclastogenic activity and SC RANKL, OPG, and CD40 expression. **A**. Images of in vitro PTH treated BM stained for TRAP to visualize OCs (top panel), and number of OCs per well (bottom panel). BM from PPR^Tcells^−/− and control mice were stimulated with PTH (1 nM) for 7 days, stained for TRAP, and OCs counted. ** = p<0.01 and *** = p<0.001 compared to vehicle. # = p<0.05 compared to the other PTH treated groups. **B** WT BMMs were cocultured with SCs isolated from PPR^T cells^ −/− and control mice treated in vivo with cPTH or vehicle. Cells were cultured for 7 days in the presence of PTH (1 nM), stained for TRAP, and OCs counted. ** = p<0.01 and *** = p<0.001 compared to vehicle. # = p<0.05 compared to the other PTH treated groups. **C**. mRNA levels of RANKL and OPG in SCs from PPR^T cells^ −/− and control mice treated in vivo with vehicle or cPTH. * = p<0.05 and ** = p<0.01 compared to the corresponding vehicle treated group. **D** mRNA levels of CD40 in SCs from PPR^T cells^ −/− and control mice treated in vivo with vehicle or cPTH. * = p<0.05 compared to the corresponding vehicle treated group. Data are Means + SEM.

To establish whether PPR signaling in T cells is required for PTH to induce OC formation in vitro, we counted the number of OCs produced by osteoclastogenic cultures of whole BM. In vitro PTH treatment caused a smaller increase in OC number in BM derived from PPR^T cells^ −/− mice, as compared to BM from control mice (Fig. 3a).

To investigate the responsible mechanism, BMMs from PPR^T cells^ −/− and control mice were cultured with RANKL and M-CSF for 7 days and OCs counted. These experiments revealed that BMMs from all genotypes generated the same number of OCs (not shown), thus excluding that silencing of PPR in T cells alters the osteoclastogenic potentials of BMMs.

We have reported that T cells potentiate PTH-induced osteoclastogenesis mainly by increasing the osteoclastogenic activity of BM SCs [11]. Thus, silencing of PPR signaling in T cells may blunt PTH-induced osteoclastogenesis by decreasing the capacity of SCs to support OC formation. To investigate this hypothesis, BMMs from intact WT mice were cultured for 7 days with SCs from either PPR^T cells^ −/− or control mice in the presence of PTH, a hormone that under these conditions promotes OC formation by targeting SCs. Cocultures containing SCs from PPR^T cells^ −/− mice produced fewer OCs, as compared to those with SCs from control mice (Fig. 3b), suggesting that the lack of PPR signaling in T cells leads to the emergence of SCs characterized by blunted sensitivity to PTH. To investigate the responsible mechanism, SCs were harvested from PPR^T cells^ −/− and control mice treated with vehicle or cPTH for 2 weeks and their production of cytokine mRNA assessed by RT-PCR. This analysis revealed that cPTH increased the expression of RANKL mRNA and lowered that of OPG mRNA in SCs from control mice but not in those from PPR^T cells^ −/− mice (Fig. 3c), thus indicating that one mechanism by which PPR signaling in T cells increases SC osteoclastogenic activity is by regulating their production of osteoclastogenic cytokines.

Silencing of PPR signaling in T cells did not alter the capacity of SCs to differentiate into OBs because SC from control and PPR^T cells^ −/− mice exhibited the same capacity to form colony forming unit-alkaline phosphatase (CFU-ALP) and bone nodules in vitro (Figure S4).

Induction of CD40 signaling in SCs by T cell-expressed CD40L is a key mechanism by which T cells sensitize SCs to PTH [11]. An important element of this regulatory loop is the capacity of PTH to upregulate the expression of CD40 in SC from T cell replete mice but not from T cell deficient mice [11]. This suggests that PTH regulates CD40 expression on SCs through T cells. Therefore, silencing of PPR signaling in T cells may blunt the osteoclastogenic activity of SCs by blocking the stimulatory effect of cPTH on CD40 expression in SCs. To investigate this hypothesis SCs were harvested from PPR^T cells^ −/− and control mice treated with vehicle or cPTH for 2 weeks and analyzed for CD40 and cytokine mRNA expression by RT-PCR. Treatment with cPTH upregulated the mRNA levels of CD40 in SCs from PPR^fl/fl^ mice, but not in SCs from PPR^T cells^ −/− mice (Fig. 3d). Confirming findings from our earlier reports [11], additional studies revealed that cPTH does not upregulate the expression of CD40L in T cells (not shown). Together, these findings suggest that one mechanism by which PPR signaling in T cells augments the osteoclastogenic activity of SCs is by upregulating their expression of CD40.

Having established that PPR signaling in T cells is required for PTH to upregulate CD40 expression in SCs, we investigated whether activation of the CD40 receptor by CD40L potentiates the SC response to PTH. To this end, WT SCs were stimulated by PTH alone or with soluble CD40L and PTH, and assayed for phosphorylated extracellular signal regulated mitogen-activated protein kinases 1 and 2 (ERK1/2), which are activated by PPR signaling. Western blot analysis revealed that combined stimulation with CD40L and PTH induced a ∼4 fold higher increase in the levels of phosphorylated ERK2 than treatment with PTH or CD40L alone (Figure S5). These findings demonstrate that SCs are synergistically activated by PPR signaling and CD40L, and attest to the relevance of CD40L/CD40 signaling in regulating the SC response to PTH.

### PPR signaling in T cells causes bone loss through increased production of TNF

**Figure 4:**
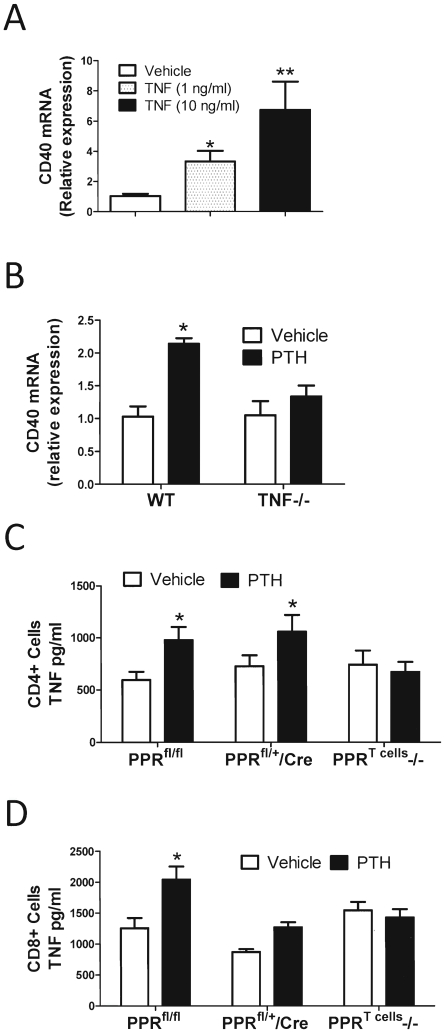
Regulation of SC CD40 expression and T cell TNF production. **A** Effect of in vitro TNF treatment on CD40 mRNA expression by SCs. **B** Effect of in vivo cPTH treatment on CD40 mRNA levels in SCs from WT and TNF−/− mice. **C** Effect of in vivo cPTH treatment on TNF mRNA and TNF protein production by CD4+ cells from PPR^T cells^ −/− and control mice. **D** Effect of in vivo cPTH treatment on TNF mRNA and TNF protein production by CD8+ cells isolated from PPR^T cells^ −/− and control mice. All data are expressed as mean ± SEM. * = p<0.05 and * = p<0.01 compared to the corresponding vehicle group. n = 6 mice per group.

We found that in vitro stimulation of SCs by TNF upregulated CD40 expression in SCs (Fig. 4a), suggesting that PTH may upregulate SC expression of CD40 through TNF. To seek in vivo confirmation, SCs were purified from WT and TNF−/− mice after the completion of 2 weeks of treatment with vehicle and cPTH, and analyzed for CD40 mRNA expression by real time RT-PCR. These studies revealed that cPTH increased CD40 mRNA levels by ∼2 folds in SCs from WT mice, while it had no effect in those from TNF−/− mice (Fig. 4b). Attesting to specificity, in vitro TNF treatment did not upregulate CD40 expression in BMMs. Moreover, in vivo cPTH treatment did not upregulate CD40 expression in BMMs from WT and TNF−/− mice (not shown).

To determine whether PTH stimulates T cell production of TNF, and if it does so via a direct effect on T cells, PPR^T cells^ −/− and control mice were treated with vehicle or cPTH for 2 weeks. Splenic CD4+ and CD8+ T cells were cultured for 48 hours in plates coated with anti CD3 and anti CD28 mAbs to induce T cell activation, and the levels of TNF in the culture media measured by ELISA. We found that cPTH increased TNF production by CD4+ (Fig. 4c) and CD8+ cells (Fig. 4d) from control mice, while there was no effect in those from PPR^T cells^ −/− mice.

By contrast, in vivo cPTH treatment did not increase the production of RANKL by CD4+ and CD8+ cells (not shown).

**Figure 5:**
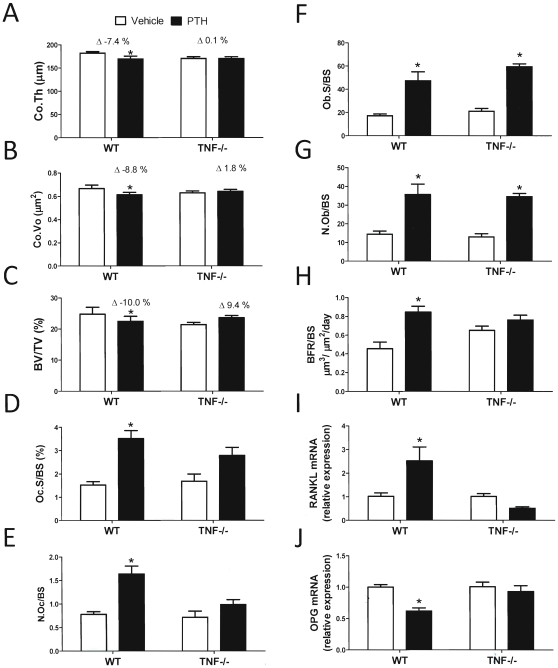
Effects of cPTH on bone structure and turnover in TNF −/− mice. **A** µCT analysis of femoral Co.Th. **B** µCT analysis of femoral Co.Vo. **C** µCT analysis of BV/TV. **D** Serum CTX levels. **E** Serum OCN levels. **F–J** Histomorphometric analysis of femoral trabecular bone. F: percentage of bone surface covered by osteoclasts (Oc.S/BS). G: number of osteoclasts per mm bone surface (N.Oc/BS). H: Bone formation rate (BFR). I: Percentage of bone surface covered by osteoblasts (Ob.S/BS). J: Number of osteoblasts per mm bone surface (N.Ob/BS). **K–L** mRNA levels of RANKL and OPG in SCs from WT and TNF−/− mice treated in vivo with vehicle or cPTH. * = p<0.05 compared to the corresponding vehicle group. n = 9–15 mice per group. Data are Means + SEM.

To determine the role of TNF in the bone loss induced by PTH, WT and TNF−/− mice were infused with cPTH for 2 weeks. Measurements by µCT revealed that cPTH caused cortical bone loss in WT but not in TNF−/− mice (Fig. 5a,b). Analysis of trabecular bone disclosed that BV/TV was significantly decreased by cPTH in WT mice. By contrast BV/TV was not significantly affected by cPTH in TNF−/− mice, although it trended toward an increase (Fig. 5c). Histomorphometric analysis of femoral trabecular bone confirmed that the number of OCs per bone surface (N.Oc/BS) and the OC surface per bone surface (Oc.S/BS), two indices of bone resorption, were increased by cPTH in WT but not in TNF −/− mice (Fig. 5d,e). By contrast, two indices of bone formation (N.Ob/BS and Ob.S/BS) were significantly augmented by cPTH in all groups (Fig. 5f,g), while a third one, BFR, was increased by iPTH treatment in WT mice and trended toward an increase in TNF−/− mice (Fig. 5h). Together, these data demonstrate that TNF is required for cPTH to increase bone turnover and cause bone loss.

To confirm that one of the mechanisms by which TNF contributes to cPTH to induce bone loss is by regulating the SC production of osteoclastogenic factors, SCs were purified from the BM of WT and TNF−/− mice treated with vehicle or cPTH, and their mRNA levels of RANKL and OPG were measured by quantitative real time RT-PCR. This analysis revealed that cPTH increased the expression of RANKL mRNA (Fig. 5i) and lowered that of OPG mRNA (Fig. 5j) in SCs from WT mice but not in those from TNF−/− mice.

**Figure 6:**
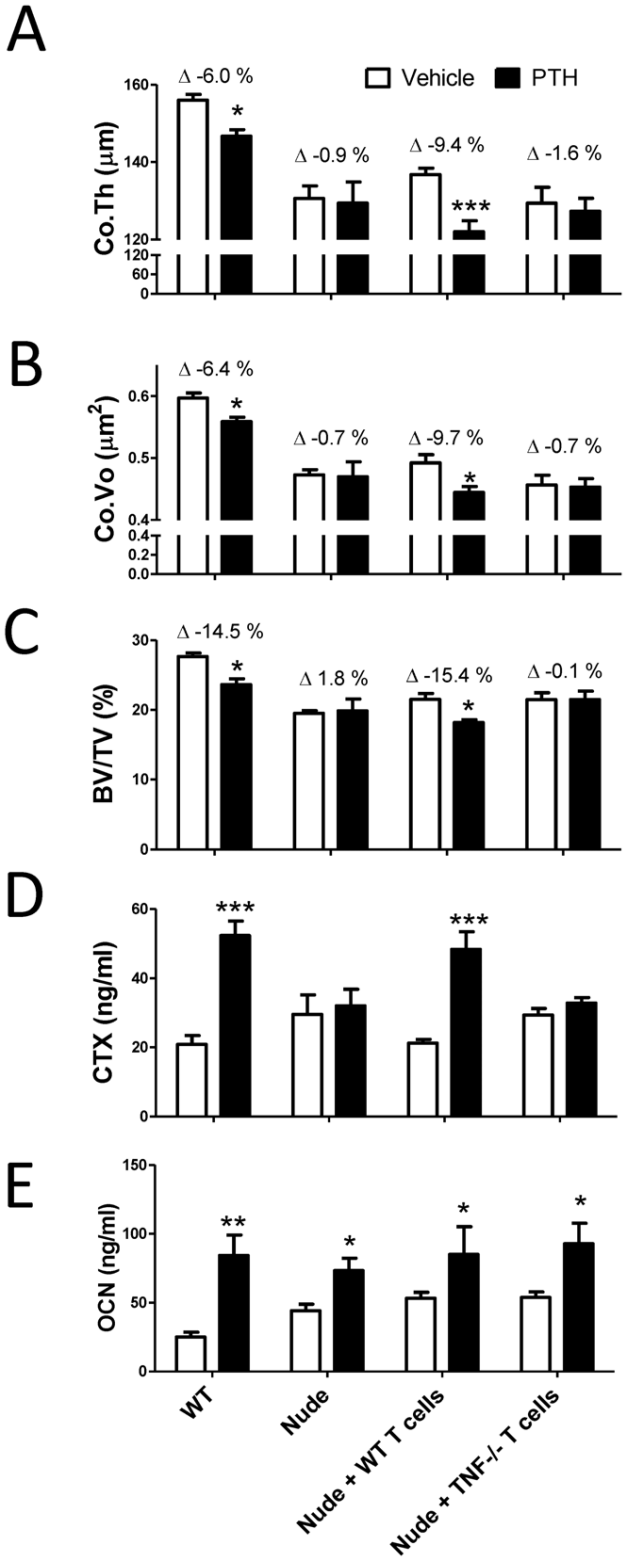
Effects of cPTH treatment in mice lacking T cell TNF production. **A** µCT analysis of femoral Co.Th. **B** µCT analysis of femoral Co.Vo. **C** µCT analysis of BV/TV. **D** Serum CTX levels. **E** Serum OCN levels. * = p<0.05, ** = p<0.01 and *** = p<0.001 compared to the corresponding vehicle treated group. n = 10–17 mice per group. Data are Means + SEM.

To investigate the specific contribution of T cell produced TNF to the bone loss induced by cPTH, nude mice were subjected to adoptive transfer of T cells purified from the spleen of WT or TNF −/− mice using established methods [11], [19], [29]. After 2 weeks, a length of time sufficient for the engraftment and the homeostatic expansion of donor T cells [11], [29], the reconstituted mice were treated with cPTH for 2 weeks. Control included WT mice and T cell deficient nude mice. Measurements of cortical and trabecular bone structure by µCT, and of biochemical indices of bone turnover revealed that cPTH caused cortical and trabecular bone loss, and an increase in serum CTX levels in WT mice and in nude mice reconstituted with WT T cells. In contrast cPTH did not induce significant changes in any of these indices in T cell deficient nude mice, or nude mice adoptively transferred with T cells from TNF−/− mice (Fig. 6a–d). However, cPTH caused a similar increase in serum osteocalcin levels in all groups (Fig. 6e). These findings demonstrate that T cell produced TNF plays a pivotal role in the bone loss and the stimulation of bone resorption induced by cPTH.

## Discussion

**Figure 7:**
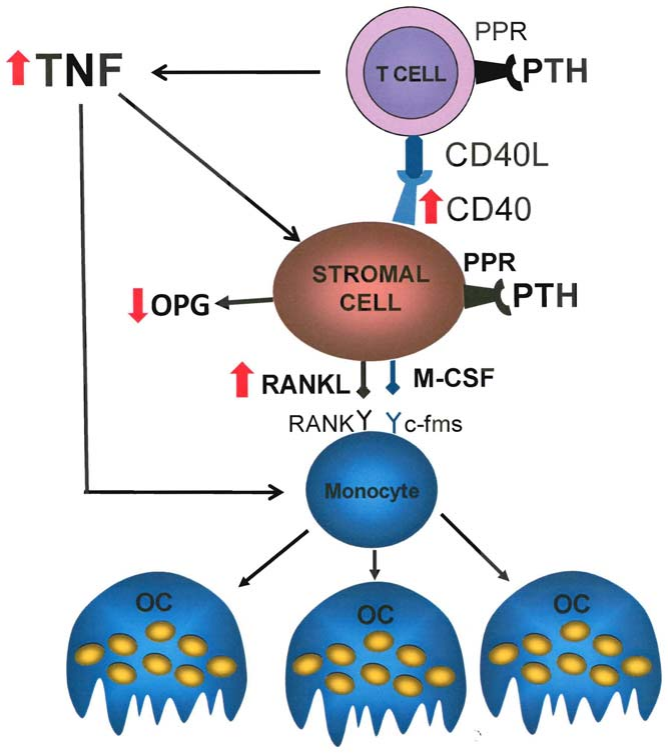
Schematic representation of the role of T cell PPR in cPTH stimulation of OC formation. PTH binding to PPR in T cells stimulates the production of TNF. This cytokine increases CD40 expression by SCs. Binding of CD40 by T cell expressed CD40L increases SC sensitivity to PTH resulting in enhanced SC production of RANKL and diminished secretion of OPG in response to PTH. T cell produced TNF further stimulates OC formation through its direct effects on maturing OC precursors. The red arrows represent the main modifications induced by activation of PPR signaling in T cells.

We report that mice lacking PPR signaling in T cells are protected against the loss of cortical and trabecular bone induced by cPTH. PPR signaling in T cells is relevant as it increases T cell production of TNF, an osteoclastogenic factor which promotes OC formation through multiple mechanisms, including direct stimulation of maturing OC precursors. In addition, T cell produced TNF augments the sensitivity of SCs to cPTH by upregulating their expression of CD40 (Fig. 7). These findings demonstrate that T cells are a direct target of cPTH which plays a pivotal role in the osteoclastogenic response to cPTH.

Three receptors for PTH have been described. The species that mediates the effects of PTH in bone is the PTH/PTHrP receptor (PPR or PTHR1), a member of a seven transmembrane-spanning domain receptor family, the G-protein coupled receptors [13], [14], [15]. An alternative PTH receptor is PTHR2, a protein expressed primarily in the central nervous system and the cardiovascular system, which is not known to play a role in bone [30], [31], [32]. Recently, a receptor specific for the carboxyl terminal PTH fragments has been proposed and is yet to be cloned [33]. The function of this receptor in bone is unknown but not relevant for the effects of PTH1-34.

We have reported that PPR mRNA is expressed in T cells and that in vitro PTH treatment increases cAMP accumulation in CD4+ and CD8+ T cells [19]. Stemming from this evidence and from the finding that the catabolic activity of cPTH is blunted in T cell-deficient mice [11], we hypothesized that T cells contribute to the bone catabolic effects of cPTH as a result of PTH binding to the PPR in T cells. To investigate this hypothesis, we genetically disrupted the PPR gene in T cells by targeted deletion of exon E1 of PPR using Cre-LoxP technology. Crossing of mice expressing an exon E1-floxed PPR allele [25] with mice that express Cre recombinase under the control of the T cell-specific promoter Lck [26], led to the generation of mice with disrupted PPR gene in T cells (PPR^T cells^ −/− mice), and intact PPR expression in other cells. The efficient and specific deletion of exon E1 of PPR in T cells was established by demonstrating a reduced exon 1-containig PPR mRNA expression in T cells from PPR^T cells^ −/− mice. The impact of PPR gene disruption on PPR function was further assessed by demonstrating attenuated PTH increased cAMP accumulation in isolated PPR^T cells^ −/− compared to controls.

At 6 weeks of age mice lacking PPR signaling in T cells have a normal bone phenotype, and normal serum calcium, phosphate and PTH levels, indicating that PPR signaling in T cells does not play a major role in bone modeling and baseline remodeling. However, at 18 weeks of age mice lacking functional PPR in T cells displayed lower BV/TV values than PPR^fl/fl^ mice, suggesting that PPR signaling in T cells contributes to the bone anabolic activity of endogenous PTH. Alternatively, the data may suggest that baseline PPR signaling per se provides bone anabolic signals that become relevant for optimal skeletal growth. Moreover, PPR^T cells^ −/− mice have a normal number of T cells that exhibit a degree of activation and proliferation similar to control T cells. However, when challenged with cPTH, PPR^T cells^ −/− mice are protected against the loss of cortical and trabecular bone observed in control mice. The resistance to cPTH-induced bone loss in PPR^T cells^ −/− mice can be explained by the reduced capacity of cPTH to stimulate bone resorption and expand the pool of resorbing OCs. Silencing of PPR signaling in T cells modified the effects of cPTH on trabecular bone from a net bone loss in control mice to bone gain in the PPR^T cells^ −/− mice. This bone gain, which is attributed to the ability of cPTH to retain its capacity to stimulate bone formation in spite of diminished resorption, is in agreement with our previous report that cPTH effects on bone formation were preserved despite the absence of T cells [11]. Consistent with the hypothesis that cPTH stimulates bone formation through a T cell independent mechanism, is the report that the osteocyte produced inhibitor of bone formation sclerostin, whose production is suppressed by cPTH [34], plays a central role in the mechanism by which continuous PPR activation increases bone formation [35]. It should be noted however that contrary to cPTH, iPTH treatment stimulates bone formation only in the presence of T cells [19]. This striking difference can be explained, at least in part, by the specific capacity of iPTH to increase T cell production of Wnt10b [19], a Wnt ligand that stimulates osteoblastogenesis and blocks OB apoptosis by activating Wnt signaling in OBs [36].

Deletion of CD40L in T cells, or lack of CD40 in SCs blunts the bone catabolic activity of cPTH by reducing the capacity of SCs to produce increased amounts of RANKL and lower amounts of OPG in response to PTH [11]. Thus T cell induction of CD40 signaling in SCs is a key mechanism by which T cells potentiate the catabolic activity of cPTH. Interestingly, cPTH regulates the CD40L/CD40 mediated cross-talk between T cells and SCs, not by regulating CD40L expression in T cells, but rather by increasing the expression of CD40 in SCs. The stimulatory activity of cPTH on SC CD40 expression is silenced in T cell deficient mice, indicating that cPTH regulates the expression of CD40 in SCs via a T cell-dependent mechanism yet to be discovered [11]. The current experiments revealed that whereas cPTH treatment increased CD40 expression in SCs derived from the BM of control mice, it failed to upregulate CD40 in SC from PPR^T cells^ −/− mice. Furthermore, similar to SCs from mice lacking T cells, CD40L, or CD40 [11], the SC from PPR^T cells^ −/−mice exhibited altered RANKL/OPG production and blunted osteoclastogenic response to cPTH. These findings indicate that T cell expressed PPR mediates the osteoclastogenic response of SCs to cPTH by upregulating CD40 expression, thereby promoting CD40L/CD40 signaling. The relevance of CD40L/CD40 signaling to SCs response to PTH was further demonstrated by finding that PTH activation of ERK2 in SCs was increased when SCs were pretreated with soluble CD40L.

It could be argued that disruption of PPR signaling in T cells does not specifically abrogates the capacity of SCs to support PTH induced osteoclastogenesis, but rather blunts the SC response to all osteoclastogenic factors. The results of the ovx experiments described herein suggest that this is not the case. In fact, we found that ovx causes similar bone loss in PPR^Tcells^ −/− and control mice treated with vehicle. Since one key mechanism by which ovx causes bone loss is by increasing SC osteoclastogenic activity [37], the data indicate that deletion of PPR in T cells does not block the increase in SC osteoclastogenic activity induced by ovx. The data rather suggest that deletion of PPR in T cells specifically blocks the SC osteoclastogenic response to PTH.

While PPR signaling in T cells regulates SC osteoclastogenic activity, silencing of T cell PPR did not alter the capacity of SCs to differentiate into OBs. This may suggest that the SCs which support OC formation are not the same cells that differentiate into OBs, the cells that form osteoid and mineralize it, a hypothesis recently proposed by others [38]. Indeed osteoclastogenic SCs express intercellular adhesion molecule-1 (ICAM-1) which is necessary for binding to OC precursors [39], while matrix-producing osteoblastic cells are ICAM-1 negative [40].

TNF is a potent osteoclastogenic cytokine which plays a pivotal role in the bone loss induced by inflammation, periodontal bone disease and estrogen deficiency [41]. Interestingly, our data show that cPTH fails to induce bone loss and stimulate bone resorption in TNF −/− mice and in nude mice adoptively transferred with TNF−/− T cells. The effects of cPTH were however restored when nude mice were adoptively transferred with WT T cells demonstrating that T cell produced TNF plays a key role in cPTH-induced bone loss. Consistently, cPTH stimulated the production of TNF by CD4+ cells and CD8+ cells from control but not PPR^Tcells^−/− mice. The diminished cPTH induced TNF production by T cells from PPR^T cells^ −/− mice establishes TNF production by T cells as a downstream effector of cPTH stimulation of PPR in T cells. These findings may help explaining the cause of increased levels of circulating TNF in patients affected by PHP [42].

TNF is known to stimulate bone resorption and causes bone loss by potentiating the osteoclastogenic activity of RANKL produced in the BM microenvironment [43], [44] and by increasing the production of RANKL by SCs and OBs via activation of the NFkB and JNK signaling pathways [45]. A novel finding is that similar to the silencing of PPR, deletion of TNF in T cells decreases the ability of cPTH to upregulate CD40 expression, and to increase the RANKL/OPG ratio in SCs. Further attesting to the capacity of TNF to upregulate CD40 expression, data show that in vitro stimulation of SCs by TNF increased the SC expression of CD40 mRNA. PPR signaling in T cells thus mediates cPTH induced CD40 expression in SCs by controlling TNF production by T cells.

Collectively, our data reveal that the effects of cPTH on bone are the result of a mechanism that involves PPR activation and TNF production in T cells. T cell produced TNF stimulates bone resorption directly by potentiating the sensitivity of maturing OCs to RANKL. In addition, TNF enhances CD40L/CD40 signaling from T cells to SCs by upregulating CD40 expression, an effect resulting in the increased capacity of SCs to support OC formation. Thus, a complex cross-talk between T cells and the osteoclastogenic machinery of the BM is central for the bone catabolic activity of cPTH.

Our study provide a new insight into the signaling integration between cells of the immune system and bone cells and the impact of such cellular interaction on bone loss. Understanding the PPR signaling in T cells and the cross-talk between T cells and SCs may thus yield novel therapeutic strategies for PTH-induced bone disease and may help understand the pathophysiology of bone loss associated with immune disorders.

## Materials and Methods

### Animals

All animal procedures were approved by the Institutional Animal Care and Use Committee of Emory University (IACUC ID # 229-2009 and 162-2008). All experiments were conducted in male or female mice of 16 weeks of age. C57BL/6 WT and TNF −/− mice were purchased from The Jackson Laboratory (Bar Harbor, Maine). All mice were maintained under non-specific pathogen free conditions and fed sterilized food and autoclaved water ad libitum.

### In vivo PTH infusion

80 µg/kg/day of hPTH1-34 (Bachem California Inc., Torrance, CA) or vehicle were delivered for 2 weeks by implanting ALZET osmotic pump model-1002 (DURECT corporation, Cupertino, CA) with a delivery rate of 0.25 µl/hr, as described [11].

### Generation of mice with T cell-specific PPR silencing

Homozygous floxed, PPR (PPR *^fl/fl^*) mice were generated as described previously [25]. Mice with T cell-specific PPR gene disruption (PPR^T cells^ −/− mice) were generated by crossing homozygous PPR*^fl/fl^* mice with *Cre* transgenic mice expressing *Cre* under the T cell specific promoter Lck. Disruption of the PPR gene was confirmed by PCR analysis of genomic DNA using forward and reverse primers specific for sequences upstream and downstream respectively, of the floxed exon E1. Disruption of the PPR gene was also confirmed by real time RT-PCR analysis of PPR mRNA using a forward primer specific for a sequence inside the floxed exon E1 and a reverse primer specific for a sequence inside exon E3.

### T cell Transfer

Nude mice were subjected to adoptive transfer of WT and TNF−/− spleen T cells via tail-vein injection of 2×10^6^ T cells purified by positive immuno-magnetic selection using MACS Microbeads (Miltenyi Biotec, Auburn, CA) coupled to non-activating anti-CD90 (Thy1.2), antibodies, as previously described [11], [19], [29]. T cells were transferred into nude mice 2 weeks before treatment to allow for engraftment and peripheral expansion of the transferred T cells, as well as the recovery of SC function. Successful T cell engraftment was confirmed by flow cytometry of splenocytes harvested at sacrifice as described previously [11], [19], [29].

### Stromal cells purification

BM SCs were purified as described previously [11]. Briefly, BM was collected from long bones by centrifugation and cultured for 5–7 days. After discarding the non adherent cells, adherent BMMs were eliminated by positive immunoselection by MACS Microbeads (Miltenyi Biotec, Auburn, CA) coupled to anti-CD11c antibody. This marker is expressed on non adherent DCs and adherent BMMs [46]. The remaining adherent cells were SCs. Cell purity was verified by histochemical staining for nonspecific esterase, a marker for cells of the monocytic/macrophage lineage. Purified SCs were >95% nonspecific esterase negative. When cultured in the presence of 50 µg/ml of ascorbic acid and 5 mM β-glycerophosphate to induce differentiation toward the osteoblastic lineage, SCs were >95% positive for alkaline phosphatase (ALP), a marker of differentiated BM SCs.

### BMMs and T cells purification and culture

BMMs and T cells were purified from the BM and spleen by positive immunoselection using MACS Microbeads (Miltenyi Biotech,) coupled to anti-CD11b, anti CD90, or anti CD4 and anti CD8 antibodies, as described [11], [19]. Cell purity was verified to be >90% by FACS.

### In vitro OC generation

BM or cultures of BMMs, SCs and T cells were cultured for 7 days in the presence of human PTH 1–34 (1 nM) to induce OC formation as described [11], [19]. The cultures were then fixed and stained for tartrate-resistant acid phosphatase (TRAP). TRAP positive cells with ≥3 nuclei were scored as OCs.

### µCT measurements of cortical and trabecular bone

µCT scanning and analysis was performed as reported previously [19] using a Scanco µCT-40 scanner (Scanco Medical, Bassersdorf, Switzerland). Bones were scanned at a resolution of 12 µm, tomographic images were obtained at conditions of 70 KV and 114 µA by collecting 500 projections. Trabecular bone volume (BV/TV) was measured in the epiphysis of the distal femurs harvested from mice sacrificed at 18 weeks of age. This region of interest was selected because at the metaphysis of 18 week old C57BL/6 mice contains little trabecular bone, a feature which prevents the detection of cPTH induced trabecular bone loss. Cortical bone volume and cortical thickness were determined by analyzing 80 slices at the mid –diaphysis of the femurs as described [11], [19].

### Quantitative bone histomorphometry

Bone histomorphometry analysis was performed at the University of Alabama at Birmingham, Center for Metabolic Bone Disease, Histomorphometry and Molecular Analysis Core Laboratory by using Bioquant Image Analysis software (R&M Biometrics, Nashville, TN), as described [11], [19]. The measurements, terminology and units used for histomorphometric analysis, were those recommended by the Nomenclature Committee of the American Society of Bone and Mineral Research [47]. Longitudinal sections (5 µm thick) were cut from Methyl Methacrylate (MMA) plastic embedded blocks along the frontal plane, using a Leica 2265 microtome, and stained with Goldner's Trichrome stain for the static measurements. Additional sections were cut at 10 µm, and left unstained for dynamic (fluorescent) measurements. For the analysis of trabecular bone, measurements were obtained in an area of the distal epiphysis of the femur spanning from 75 µm from the growth plate to the endocortical edge of the epiphysis, as previously described [48]. N.Ob/BS (the number of OBs per mm bone surface), ObS/BS (the percentage of bone surface covered by OBs), N.OC/BS (the number of OCs per mm bone surface), OcS/BS (the percentage of BS occupied by OCs) and bone formation rate (BFR) were measured.

### Measurement of serum markers of bone turnover

Serum markers of bone turnover have been measured as described previously [11], [19]. Serum CTX, a marker of bone resorption, was measured by a rodent specific ELISA assay (Immunodiagnostic Systems, Scottsdale AZ). Serum osteocalcin (OCN), a specific marker for bone formation, was measured using Rat-MID™ Osteocalcin ELISA kit (Nordic Bioscience Diagnostics A/S, Herlev, Denmark).

### Measurement of Serum Calcium, inorganic phosphate and PTH

Serum calcium was measured by a colorimetric kit, Calcium LiquiColor (Stanbio Laboratory, Boerne, TX). Serum levels of endogenous PTH (1–84) were measured by ELISA using a commercial kit (Immutopics, Inc., San Clemente, CA). Inorganic phosphate was measured using Phosphorous Liqui-UV kits (Standbio, Boerne, TX) as per manufacturer's instructions.

### TNF measurement by ELISA

Splenic CD4+ and CD8+ T cells were purified from PPR^T cells^ −/− and control mice treated with vehicle or cPTH for 2 weeks. Cells were then cultured for 48 hours in plates coated with anti CD3 and anti CD28 mAbs. TNF protein levels in the culture media were measured by ELISA.

### Real-Time RT-PCR

RANKL, OPG, CD40, and PPR mRNA were quantitated by real-time PCR using a GeneAmp 7000 system (PE Biosystems) and changes in relative gene expression between groups was calculated using the 2–ΔΔCT method with normalization to 18S rRNA as previously described [49]. All primers for real time PCR were designed by using Primer Express® Software v2.0 (PE Biosystems). The sequence of primers used were: 5′- ATTCGAACGTCTGCCCTATCA -3′ (forward) and 5- GTCACCCGTGGTCACCATG -3′ (reverse) for 18 s, 5′- CCTGATGAAAGGAGGGAGCA -3′ (forward) and 5′- TGGAATTCAGAATTGCCCGA -3′ (reverse) for RANKL, and 5′-CTTGGGTCTGTTGCTTGGTGA-3′ (forward) and 5′-GCCGCTTCCTTA CACACCAG-3′ (reverse) for OPG, 5′-GCAGGAGCGGCAGGTGACAG-3′ (forward) and 5′-ACGCCGCAGCAAGACGACTC-3′ (reverse) for CD40, 5′-ACAAAGGGTGGACGCCAGCA-3′ (forward, inside PPR exon E1) and 5′-GCGGTCGCAGCGTCTGTAGG -3′ (reverse, inside PPR exon E3), 5′-TAATGCTCAGCGCCCCGT-3′ (forward, upstream of PPR exon E1) and 5′-AGCAGCAGACGCCGAGGCGA-3′ (reverse, downstream of PPR exon E1) for PPR. Amplification reactions were performed in 25 µl containing 0.5 µM of primers, dNTPs (0.2 mM each) in PCR buffer and 0.03 U Taq polymerase along with SYBR-green (Molecular Probes, Eugene, OR). Dissociation curves revealed a single product in all cases.

### Measurement of cAMP accumulation in T cells

cAMP was measured by radioimmunoassay as described previously [19]. Briefly, CD4+ and CD8+ spleen T cells were pre-treated with 1 mM IBMX (Sigma-Aldrich Biochemicals) for 1 hour, and then treated with either 50 nM PTH (1–34), or vehicle for 10 minutes. cAMP was measured using a commercial radioimmunoassay kit (PerkinElmer,Shelton CT) as per kit instructions. Protein concentration was measured using the Bio-Rad Dc (Bio-Rad) protein assay kit. cAMP concentrations were expressed as pMol per mg protein.

### Statistical Analysis

For each outcome, a two-way analysis-of-variance was applied that included the main effects for animal strain and treatment plus the statistical interaction between animal strain and treatment. When the statistical interaction between animal strain and treatment group was not statistically significant (P>0.05) nor suggestive of an important interaction (P>0.10) p values for the main effects tests were reported. When the statistical interaction was statistically significant or suggestive of an important interaction then t-tests were used to compare the differences between the treatment means for each animal strain, applying the Bonferroni correction for multiple comparisons. The response studies shown in figure 4a were analyzed by one-way ANOVA.

## Supporting Information

Figure S1 Serum calcium, inorganic phosphate, PTH, BMD and T cell function in PPR ^Tcells−/−^ and control mice. A Serum levels of calcium, inorganic phosphate and intact PTH at 6 weeks of age. B Femoral BMD (Mean ± SEM) at 6 weeks of age was measured in anesthetized mice using a PIXImus2 bone densitometer (GE Medical System, Lunar, Madison, WI). n = 20 mice per group. C Splenocytes were stained with APC anti-mouse CD4, PerCP anti-mouse CD8, and analyzed by FACS. Data are expressed as percentage. D Splenocytes were stimulated with plate bound anti-CD3 and anti-CD28 mAbs for 24 hours, and stained with FITC anti-mouse CD25. The cells were gated on CD4 and CD8 and analyzed by FACS for expression of the activation marker CD25. E CD90+ T cells were purified from the spleen of untreated mice, stimulated with plate bound anti-CD3 and anti-CD28 mAbs for 48 hours at the indicated doses and pulsed with [3H] thymidine for the last 18 hours to assess their proliferation. Data were analyzed by one-way ANOVA and expressed as CPM.
(6.80 MB TIF)

Figure S2 Effects of cPTH on bone structure and turnover in female mice. A–C. Cortical and trabecular bone analysis µCT. D–E Serum markers of bone turnover. CTX is a marker of resorption. OCN is a marker of formation. F–I Histomorphometric analysis femoral trabecular bone. F: percentage of bone surface covered by osteoclasts (OcS/BS). G: number of osteoclasts per mm bone surface (N.Oc/BS). H: Percentage of bone surface covered by osteoblasts (ObS/BS). I: Number of osteoblasts per mm bone surface (N.Ob/BS). * = p<0.05, ** = p<0.01 and *** = p<0.001 compared to the corresponding vehicle treated group. # = p<0.05 compared to the corresponding PPR^fl/fl^ mice. n = 12–20 mice per group for µCT and serum measurements. n = 10 mice per group for bone histomorphometry. Data are Means ± SEM.
(1.91 MB TIF)

Figure S3 Effects of cPTH treatment on cortical and trabecular bone volume in ovariectomized mice. PPR^fl/fl^ and PPR^T cells −/−^ mice were ovariectomized (ovx) at 16 weeks of age, treated with vehicle or cPTH for 2 weeks, and sacrificed. Femoral BMD was measured in vivo by DEXA at baseline and at 2 weeks. Femurs were harvested and analyzed by µCT. A Femoral BMD. B cortical thickness (Co.Th), C cortical volume (Co.Vo), and trabecular bone volume (BV/TV). * = p<0.05 compared to the corresponding vehicle treated group. # = p<0.05 compared to the corresponding PPR^T cells −/−^ mice. Data are Means ± SEM.
(2.96 MB TIF)

Figure S4 CFU-ALP and mineralization nodules formation in PPR^Tcells−/−^ and control mice. Top panel. Whole BM from PPR^T cell −/−^ and control mice were cultured for 7 days to assess the formation CFU-ALP. Bottom panel: Whole BM was cultured for 14 days in α-MEM supplemented with 10% FBS, 1% penicillin-streptomycin, and 10 mM of β-glycerophosphate for 2 weeks, and stained with AgNO3 by the Von Kossa method to detect phosphate deposits in bone nodules. The panel shows representative quadruplicate wells per group.
(6.35 MB TIF)

Figure S5 Effect of combined treatment with CD40L and PTH on the SC level of phosphorylated ERK1/2. SCs were purified from intact WT mice and plated at 250,000 cells/well on 12 well plates. After an overnight incubation SCs were serum starved for 1 hour followed by treatment with recombinant CD40L (100 ng/ml) for 5 min. PTH (5 nM) was then added and incubation continued for 10 min. Cells were rinsed twice with ice-cold PBS and collected in SDS lyses buffer. Equal amounts of sample lysates were loaded and proteins were analyzed on 8% SDS polyacrylamide gel electrophoresis. The samples were transferred onto nitrocellulose membrane and blotted and reblotted with antibodies against phospho-ERK1/2 (P-ERK1/2), total-ERK1/2, and β-actin and appropriate peroxidase-conjugated secondary antibodies. Detection was performed using chemiluminescence and the membranes were exposed to a film for 0.5 min. Densitometric quantification of P-ERK2 and Total- ERK2 protein bands was performed. Data are expressed as fold change compared to vehicle.
(3.87 MB TIF)
