# Supplementary figures and images for: Disruption of PTH Receptor 1 in T Cells Protects against PTH-Induced Bone Loss
Source: PLoS One. 2010 Aug 20;5(8):e12290. doi: 10.1371/journal.pone.0012290 (PMC2924900; doi:10.1371/journal.pone.0012290)

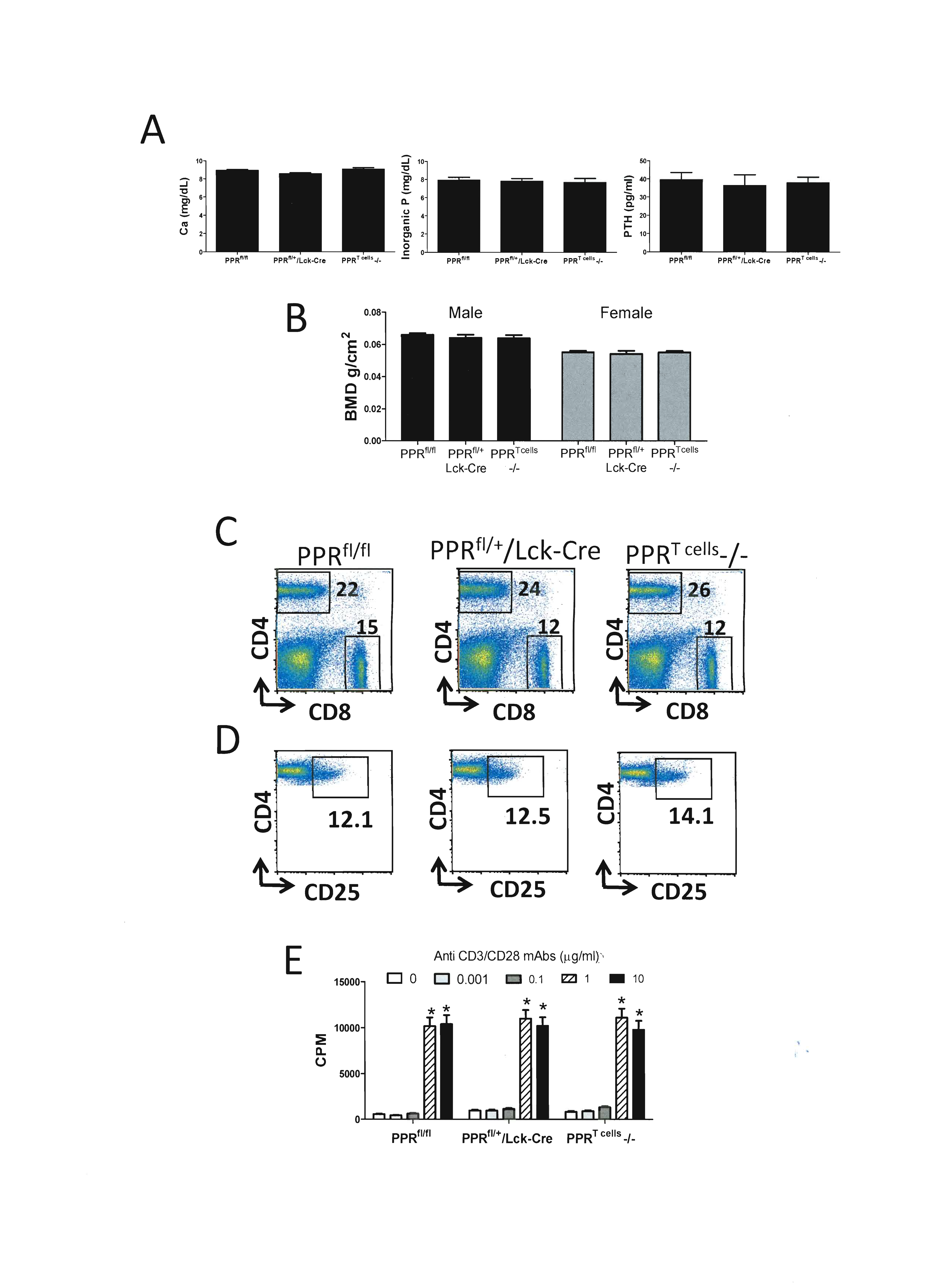

Supplement: Figure S1 — Serum calcium, inorganic phosphate, PTH, BMD and T cell function in PPR Tcells−/− and control mice. A Serum levels of calcium, inorganic phosphate and intact PTH at 6 weeks of age. B Femoral BMD (Mean ± SEM) at 6 weeks of age was measured in anesthetized mice using a PIXImus2 bone densitometer (GE Medical System, Lunar, Madison, WI). n = 20 mice per group. C Splenocytes were stained with APC anti-mouse CD4, PerCP anti-mouse CD8, and analyzed by FACS. Data are expressed as percentage. D Splenocytes were stimulated with plate bound anti-CD3 and anti-CD28 mAbs for 24 hours, and stained with FITC anti-mouse CD25. The cells were gated on CD4 and CD8 and analyzed by FACS for expression of the activation marker CD25. E CD90+ T cells were purified from the spleen of untreated mice, stimulated with plate bound anti-CD3 and anti-CD28 mAbs for 48 hours at the indicated doses and pulsed with [3H] thymidine for the last 18 hours to assess their proliferation. Data were analyzed by one-way ANOVA and expressed as CPM. (6.80 MB TIF) [file pone.0012290.s001.tif]

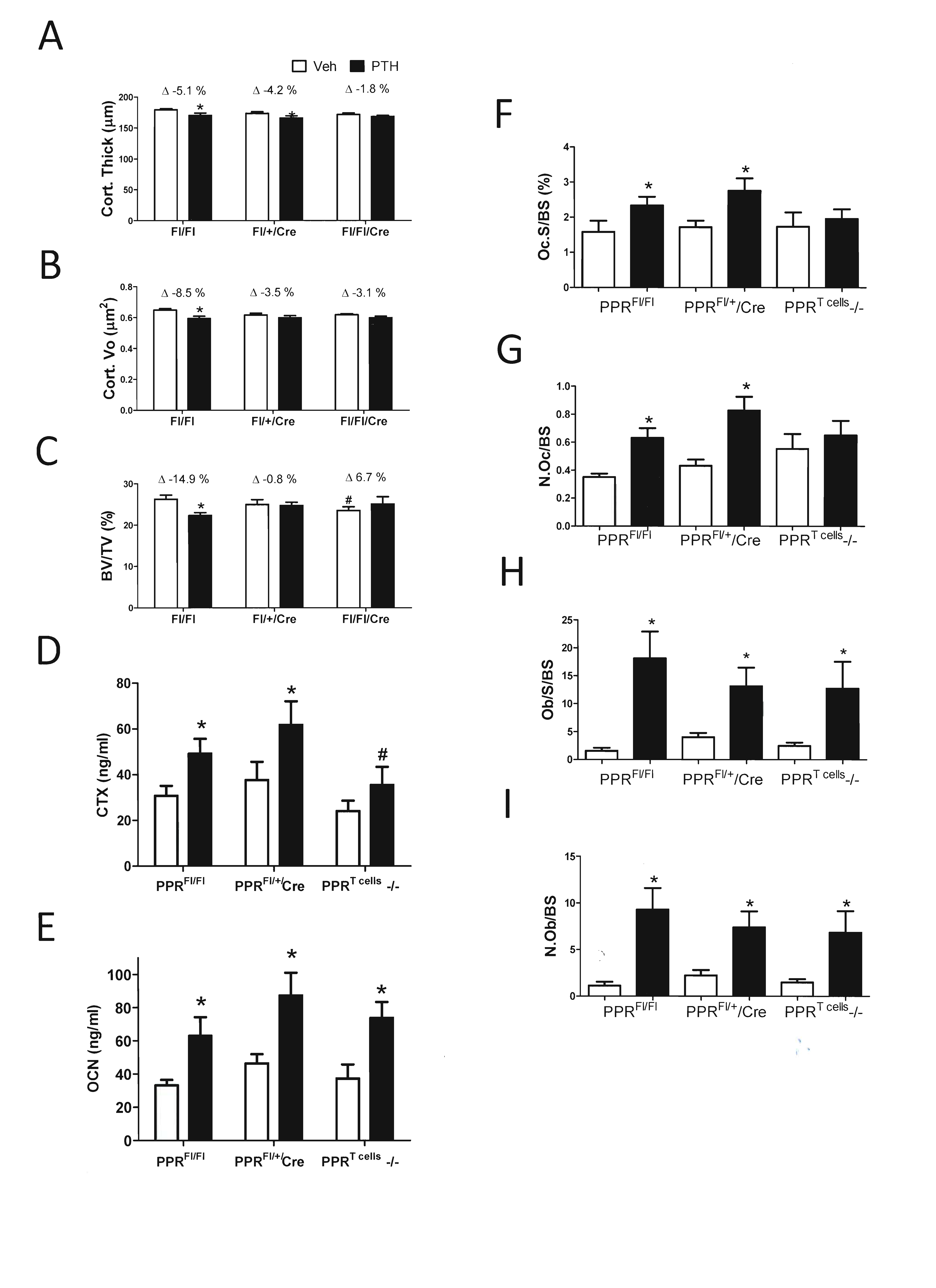

Supplement: Figure S2 — Effects of cPTH on bone structure and turnover in female mice. A–C. Cortical and trabecular bone analysis µCT. D–E Serum markers of bone turnover. CTX is a marker of resorption. OCN is a marker of formation. F–I Histomorphometric analysis femoral trabecular bone. F: percentage of bone surface covered by osteoclasts (OcS/BS). G: number of osteoclasts per mm bone surface (N.Oc/BS). H: Percentage of bone surface covered by osteoblasts (ObS/BS). I: Number of osteoblasts per mm bone surface (N.Ob/BS). * = p<0.05, ** = p<0.01 and *** = p<0.001 compared to the corresponding vehicle treated group. # = p<0.05 compared to the corresponding PPRfl/fl mice. n = 12–20 mice per group for µCT and serum measurements. n = 10 mice per group for bone histomorphometry. Data are Means ± SEM. (1.91 MB TIF) [file pone.0012290.s002.tif]

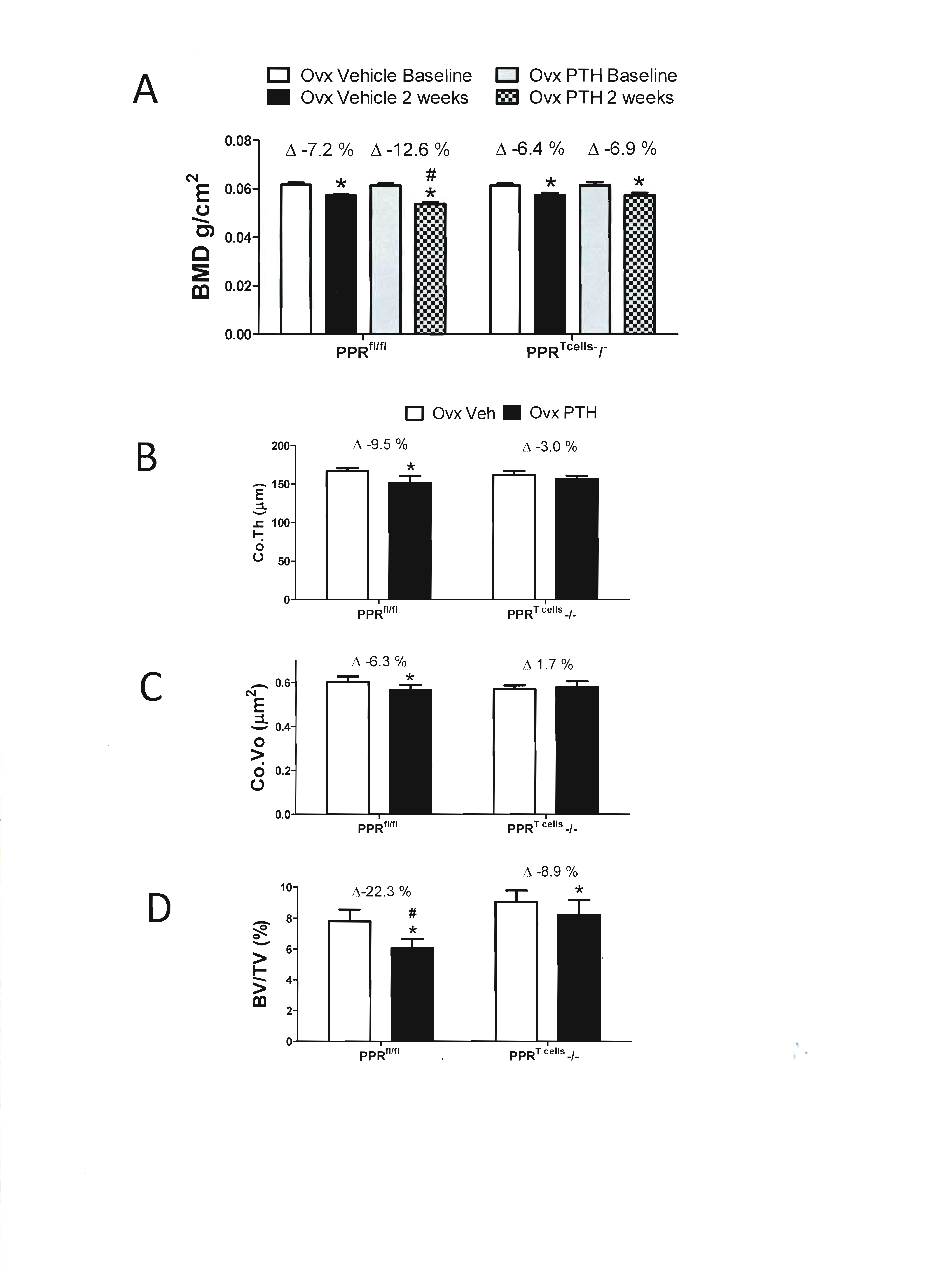

Supplement: Figure S3 — Effects of cPTH treatment on cortical and trabecular bone volume in ovariectomized mice. PPRfl/fl and PPRT cells −/− mice were ovariectomized (ovx) at 16 weeks of age, treated with vehicle or cPTH for 2 weeks, and sacrificed. Femoral BMD was measured in vivo by DEXA at baseline and at 2 weeks. Femurs were harvested and analyzed by µCT. A Femoral BMD. B cortical thickness (Co.Th), C cortical volume (Co.Vo), and trabecular bone volume (BV/TV). * = p<0.05 compared to the corresponding vehicle treated group. # = p<0.05 compared to the corresponding PPRT cells −/− mice. Data are Means ± SEM. (2.96 MB TIF) [file pone.0012290.s003.tif]

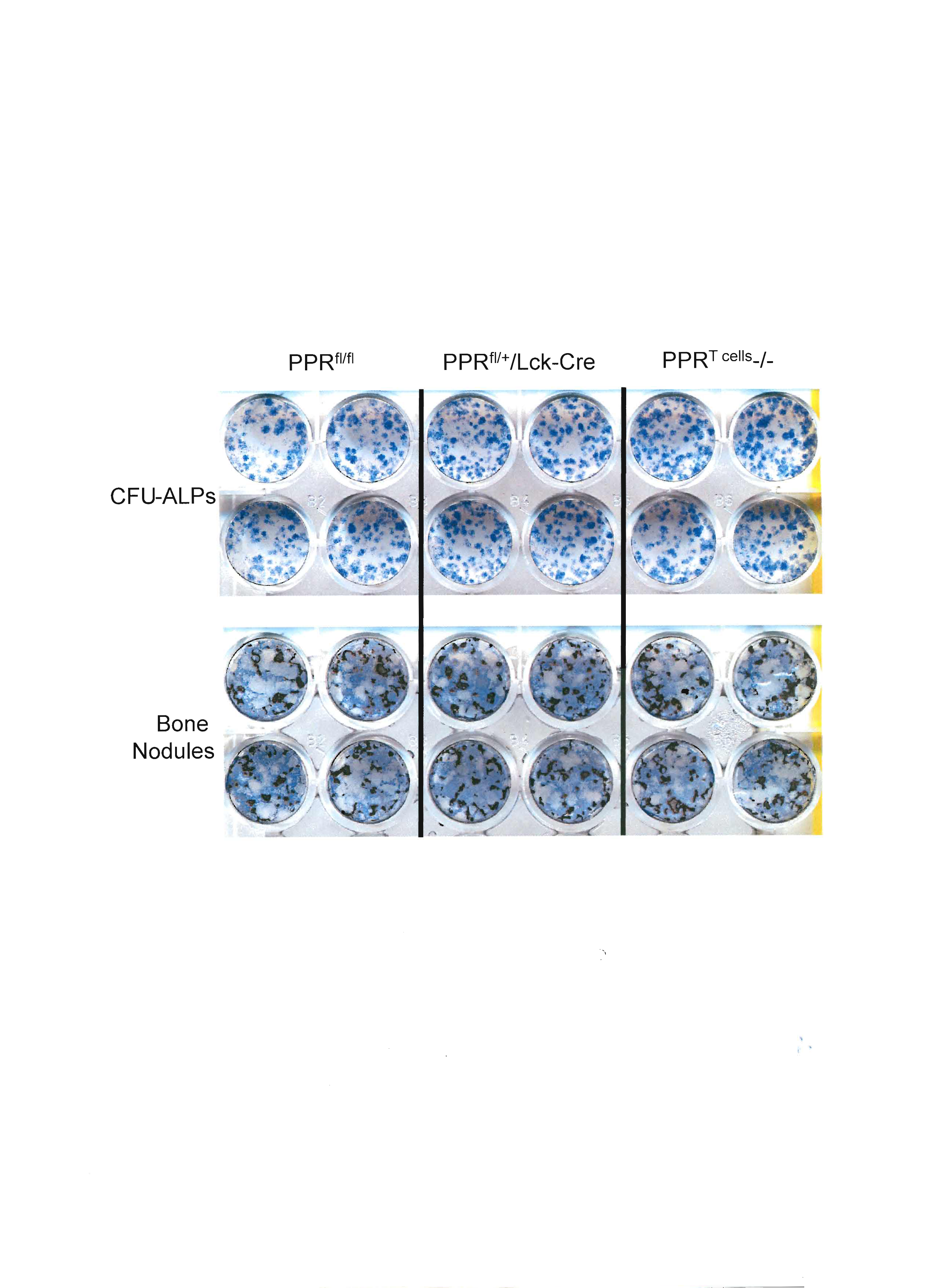

Supplement: Figure S4 — CFU-ALP and mineralization nodules formation in PPRTcells−/− and control mice. Top panel. Whole BM from PPRT cell −/− and control mice were cultured for 7 days to assess the formation CFU-ALP. Bottom panel: Whole BM was cultured for 14 days in α-MEM supplemented with 10% FBS, 1% penicillin-streptomycin, and 10 mM of β-glycerophosphate for 2 weeks, and stained with AgNO3 by the Von Kossa method to detect phosphate deposits in bone nodules. The panel shows representative quadruplicate wells per group. (6.35 MB TIF) [file pone.0012290.s004.tif]

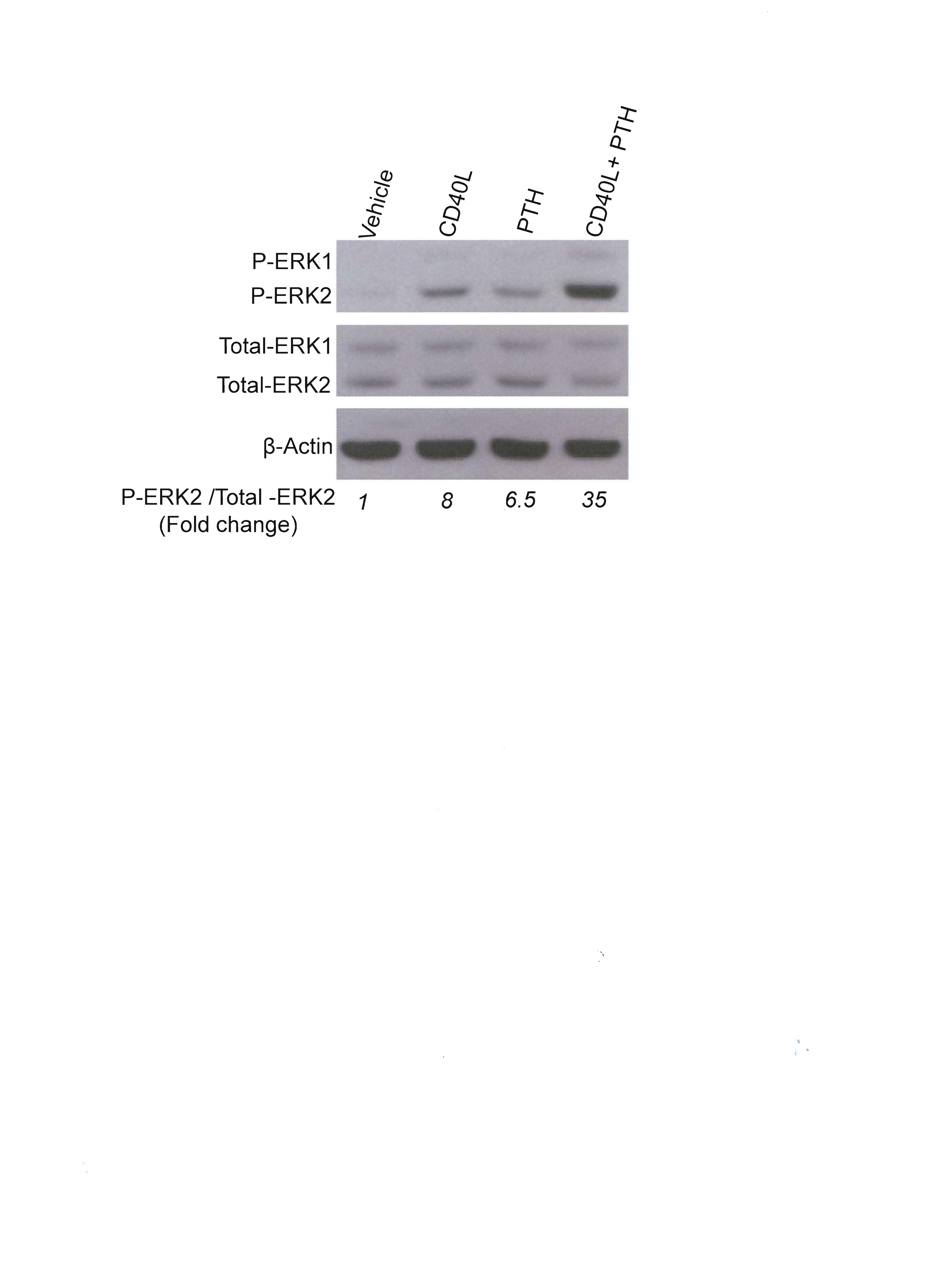

Supplement: Figure S5 — Effect of combined treatment with CD40L and PTH on the SC level of phosphorylated ERK1/2. SCs were purified from intact WT mice and plated at 250,000 cells/well on 12 well plates. After an overnight incubation SCs were serum starved for 1 hour followed by treatment with recombinant CD40L (100 ng/ml) for 5 min. PTH (5 nM) was then added and incubation continued for 10 min. Cells were rinsed twice with ice-cold PBS and collected in SDS lyses buffer. Equal amounts of sample lysates were loaded and proteins were analyzed on 8% SDS polyacrylamide gel electrophoresis. The samples were transferred onto nitrocellulose membrane and blotted and reblotted with antibodies against phospho-ERK1/2 (P-ERK1/2), total-ERK1/2, and β-actin and appropriate peroxidase-conjugated secondary antibodies. Detection was performed using chemiluminescence and the membranes were exposed to a film for 0.5 min. Densitometric quantification of P-ERK2 and Total- ERK2 protein bands was performed. Data are expressed as fold change compared to vehicle. (3.87 MB TIF) [file pone.0012290.s005.tif]
